# Supplementary material for: Adverse Events of Percutaneous Microaxial Left Ventricular Assist Devices—A Retrospective, Single-Centre Cohort Study
Source: J Clin Med. 2021 Aug 20;10(16):3710. doi: 10.3390/jcm10163710 (PMC8396891; doi:10.3390/jcm10163710)
Supplement: Supplementary file 1 [file jcm-10-03710-s001.zip › jcm-1288047-supplementary.pdf]

Supplementary Table S1: Adverse Events stratified by sex

**Outcome and adverse events**	**All patients** n (%), median (IQR), n=281	**Men** n (%), median (IQR), n=210	**Women** n (%), median (IQR), n=71
Bleeding *			
No bleeding	83 (29.5)	70 (33.3)	13 (18.3)
BARC types 1&2	103 (36.7)	80 (38.1)	23 (32.4)
BARC type 3	175 (62.3)	118 (56.2)	57 (80.3)
BARC type 4	24 (8.5)	13 (6.2)	11 (15.5)
BARC type 5	5 (1.8)	3 (1.4)	2 (2.8)
Number of all patients needing transfusions †	163 (58)	112 (53.3)	51 (71.8)
Number of RBC transfusions, units (n=164, median	4 (2-12)	4 (2-11)	6 (2-17)
Stroke	21 (7.5)	16 (7.6)	5 (7)
Stroke during Impella® therapy	12 (4.3)	10 (4.8)	2 (2.9)
Ischemic complications (non-central nervous)‡	56 (19.9)	35 (16.7)	21 (29.6)
Intestinal ischemia	25 (8.9)	17 (8.1)	8 (11.3)
Limb ischemia	36 (12.8)	22 (10.5)	14 (19.7)
Thrombotic complications (jugular venous)	3 (1.1)	2 (1)	1 (1,4)
Worsening of the valve function‡	18 (6.4)	14 (6.7)	4 (5.6)
Aortic valve	6 (2.1)		
Mitral valve	14 (5)		
New posterior mitral valve chordal rupture	4 (1.4)	4 (1.9)	0
Hemorrhagic shock	9 (3.2)	6 (2.9)	3 (4.3)
Associated with cardiac surgery (included in BARC	4 (1.4)	4 (1.9)	0
All vascular complications (aneurysms, dissections,	12 (4.3)	9 (4.3)	3 (4.2)
Vascular complications without surgery	4 (1.4)		

BARC: Bleeding Academic Research Consortium, RBC: red blood cells, IQR: interquartile range, LVEF: left ventricular ejection fraction. * During the ICU stay, multiple types possible. † During Impella® therapy. ‡ Multiple factors possible.

Supplementary Table S2: Adverse events stratified by age categories

**Outcome and adverse events**	**All patients** n (%), median (IQR), n=281	**Age <45 years** n (%), median (IQR), n=16	**Age 45-65 years** n (%), median (IQR), n=126	**Age >65 years** n (%), median (IQR), n= 139
Bleeding *				
No bleeding	83 (29.5)	4 (25)	45 (35.7)	34 (24.5)
BARC types 1&2	103 (36.7)	6 (37.5)	50 (39.7)	47 (33.8)
BARC type 3	175 (62.3)	12 (75)	63 (50)	100 (71.9)
BARC type 4	24 (8.5)	1 (6.3)	8 (6.4)	15 (10.8)
BARC type 5	5 (1.8)	1 (6.3)	2 (1.6)	2 (1.4)
Number of all patients needing transfusions †	163 (58)	12 (75)	61 (48.4)	90 (64.8)
Number of RBC transfusions, units (n=164, median [IQR])	4 (2-12)	9 (5-13)	5 (2-12)	4 (2-12)
Stroke	21 (7.5)	2 (12.5)	10 (7.9)	9 (6.5)
Stroke during Impella® therapy	12 (4.3)	0 (0)	6 (4.8)	6 (4.3)
Ischemic complications (non-central nervous system)‡	56 (19.9)	4 (25)	30 (23.8)	22 (15.8)
Intestinal ischemia	25 (8.9)	2 (12.5)	14 (11.1)	9 (6.5)
Limb ischemia	36 (12.8)	2 (12.5)	18 (14.3)	16 (11.5)
Thrombotic complications	3 (1.1)	0 (0)	2 (1.6)	1 (0.7)
Worsening of the valve function‡	18 (6.4)	1 (6.3)	12 (9.5)	5 (3.6)
Aortic valve	6 (2.1)			
Mitral valve	14 (5)			
New posterior mitral valve chordal rupture	4 (1.4)	0 (0)	3 (2.4)	1 (0.7)
Hemorrhagic shock	9 (3.2)	1 (6.3)	4 (3.2)	4 (2.9)
Associated with cardiac surgery	4 (1.4)	0 (0)	1 (0.8)	3 (2.2)
All vascular complications	12 (4.3)	3 (18.8)	4 (3.2)	5 (3.6)
Vascular complications without	4 (1.4)			

BARC: Bleeding Academic Research Consortium, RBC: red blood cells, IQR: interquartile range, LVEF: left ventricular ejection fraction. * During the ICU stay, multiple types possible. † During Impella® therapy. ‡ Multiple factors possible.
